# Mitochondrial respiratory inhibition by 2,3‐butanedione monoxime (BDM): implications for culturing isolated mouse ventricular cardiomyocytes

**DOI:** 10.14814/phy2.12606

**Published:** 2016-01-05

**Authors:** Andrew R. Hall, Derek J. Hausenloy

**Affiliations:** ^1^ The Hatter Cardiovascular InstituteInstitute of Cardiovascular ScienceUniversity College London Hospital & Medical SchoolLondonUK; ^2^ Cardiovascular and Metabolic Disorders ProgramDuke‐National University of SingaporeSingaporeSingapore; ^3^ National Heart Research Institute SingaporeNational Heart Centre SingaporeSingaporeSingapore

**Keywords:** BDM, murine cardiomyocytes, respiration

## Abstract

Experiments in isolated ventricular cardiomyocytes have greatly facilitated the study of cellular and subcellular physiology in the heart. However, the isolation and culture of high‐quality adult murine ventricular cardiomyocytes can be technically challenging. In most experimental protocols, the culture of viable adult murine cardiomyocytes for prolonged time periods is achieved with the addition of the myosin II ATPase inhibitors blebbistatin and/or 2,3‐butanedione monoxime (BDM). These drugs are added to increase cell viability and life span by inhibiting spontaneous cardiomyocyte contraction, thereby preventing calcium overload, cell hypercontracture, and cell death. While the addition of BDM has been reported to prolong the life span of isolated adult murine cardiomyocytes, it is also associated with several off‐target effects. Here, we report a novel off‐target effect, in which BDM inhibits mitochondrial respiration by acting directly on the electron transport chain to reduce cell viability. In contrast, when cells were cultured with blebbistatin alone, cells survived for longer, and no metabolic off‐target effects were observed. Based on these novel observations, we recommend that culture media for isolated mouse ventricular cardiomyocytes should be supplemented with blebbistatin alone, as BDM has the potential to affect mitochondrial respiration and cell viability, effects which may impact adversely on subsequent experiments.

## Introduction

The ability to undertake experiments in isolated murine ventricular cardiomyocytes have greatly facilitated the study of cellular and subcellular physiology in the heart (ranging from functional parameters such as ion channel activity, calcium handling, and contractile function, to signaling cascades and posttranslational modifications). Cardiac‐specific and inducible promoters have allowed genetic knockout and knockin mice to be used to investigate protein function specifically in the heart.

High‐quality isolated ventricular cardiomyocytes are required for these experimental studies to ensure that reproducible and reliable data are obtained (reviewed in Louch et al. 2011). While adult rat ventricular cardiomyocytes are fairly robust and survive ex vivo for several days, the isolation of adult murine ventricular cardiomyocytes is more challenging as the cells are more fragile and the results of isolation are less consistent and sensitive – the cell often undergoing rigor contracture and cell death within 24 h of isolation. In order to prolong adult murine ventricular cardiomyocyte viability, isolation buffers and culture media are often supplemented with inhibitors of myosin II ATPase such as 2,3‐butanedione monoxime (BDM) or blebbistatin (Kivisto et al. 1995; Farman et al. 2008; Kabaeva et al. 2008). Although BDM inhibits cardiac contraction, it also has multiple off‐target effects including altering L‐type Ca^2+^ channel activity, SR Ca^2+^ load (Gwathmey et al. 1991), and cardiac action potential characteristics and conduction (Kettlewell et al. 2004; Lou et al. 2012). On the other hand, blebbistatin has been reported to be more specific, with fewer off‐target effects than BDM (Farman et al. 2008), with little effect of cardiac action potential duration and characteristics (Fedorov et al. 2007; Lou et al. 2012), L‐type Ca^2+^ channel activity (Dou et al. 2007), or Ca^2+^ transient propagation (Farman et al. 2008).

In the current study, we make the novel observation that culturing isolated adult murine ventricular cardiomyocytes with BDM also causes significant inhibition of mitochondrial respiration, resulting in worse cell viability. In contrast, culturing cells with blebbistatin prolonged cell viability, with no effects on mitochondrial respiration.

## Methods

### Ethical statement

All experiments were performed according to Home Office (UK) guidelines under the 1986 Animal (Scientific Procedures) Act with University College London Ethics Committee approval.

### Isolation of adult murine ventricular cardiomyocytes

Cardiomyocytes were isolated from C57/BL6 mice aged between 8 and 10 weeks (Ibarra et al. 2013). Briefly, mice were anesthetized using an intraperitoneal injection of 100 U pentobarbital and 30 U heparin. Hearts were excised into ice‐cold perfusion buffer and cannulated via the aorta before retrograde perfusion on a modified Langendorff‐system at 37°C. Perfusion buffer consisted of (in mmol/L): NaCl 113, KCl 4.7, KH_2_PO_4_ 0.6, Na_2_HPO_4_ 0.6, MgSO_4_‐7H_2_0 1.2, NaHCO_3_ 12, KHCO_3_ 10, Hepes Na salt 0.922, Taurine 30, BDM 10, Glucose 5.5, and was sterile filtered prior to use. To clear the coronaries of residual blood, hearts were perfused with the perfusion solution for 5 min. Following this, the perfusate was switched to digestion buffer for 20 min, which consisted of 30 mL perfusion buffer containing 5 mg Liberase (Roche, UK) and 12.5 μmol/L CaCl_2_. Digestion buffer was collected and recirculated appropriately. After 20 min of perfusion the heart had become enlarged, paler, and soft to touch. The heart was gently removed from the cannula, and in 4 mL of digestion buffer it was teased apart, initially by tweezers and then by gentle pipetting in a Pasteur pipette. The heart cells were transferred into a new 15 mL Falcon and the solution topped up to 10 mL with stop buffer 1 (1 mL FBS per 10 mL perfusion buffer). Cells were allowed to pellet for 10 min, after which the supernatant was removed and cells resuspended in increasing concentrations of Ca^2+^ (62 μmol/L, 212 μmol/L, and 1 mmol/L). Cells were then resuspended in either M199 supplemented with l‐carnitine (2 mmol/L), creatine (5 mmol/L), taurine (5 mmol/L), and penicillin (100 IU/mL), streptomycin (100 IU/mL) (for cell culture experiments) or unbuffered DMEM supplemented with 4 mmol/L glutamine, 5 mmol/L glucose, 200 μmol/L carnitine, 100 μmol/L Na pyruvate, 100 μmol/L BSA‐palmatate and 10 mmol/L HEPES, pH 7.4 (for whole cell respiration experiments) (Readnower et al. 2012).

### Culture of isolated adult murine ventricular cardiomyocytes

Cardiomyocytes were plated onto prelaminated six‐well plates suspended in M199 (Invitrogen, UK) media with either no supplementation, or the addition of either 25 μmol/L blebbistatin, 9.9 μmol/L BDM (equivalent to 1 mg/mL), or both 25 μmol/L blebbistatin and 9.9 μmol/L BDM. These concentrations of blebbistatin and BDM have been typically used to prolong cardiomyocyte viability for prolonged culture (Liao and Jain 2007; Kabaeva et al. 2008; Louch et al. 2011; Readnower et al. 2012). The media was changed after 2 h and images taken 2, 24, and 48 h later. Where appropriate, cell death was assessed through the addition of propidium iodide just prior to imaging. Spontaneous contractions were quantified after 2 h postplating by video imaging for 1 min. The number of spontaneous contractions in the set field of view were counted and recorded. Equal cell density was assumed between conditions and experimental replicates.

### Measurement of whole cell respiration

Whole cell cardiomyocyte respiration was measured using an Oxytherm (Hansatech, UK). Isolated cardiomyocytes were resuspended in 600 *μ*L unbuffered DMEM, which was either unsupplemented or supplemented with 25 μmol/L blebbistatin, 9.9 μmol/L BDM, or both 25 μmol/L blebbistatin and 9.9 μmol/L BDM. Basal respiration was established at 30°C, after which oligomycin (1 *μ*g/mL), FCCP (2 μmol/L), antimycin A (5 μmol/L), and rotenone (2 μmol/L) were added. Respirometry was normalized to cell protein concentration (samples were sonicated and protein content assessed by Bradford assay). For the Bradford assay, BSA protein standards were prepared at the following concentrations (0, 0.4, 0.8, 1.2, 1.6, and 2 mg/mL) from a 2 mg/mL stock solution. Samples were diluted 1:10 in water and pipetted out into a 96‐well plate, before the addition of 200 *μ*L of bicinchoninic acid and copper(II) sulfate in a 50:1 ratio. Sample absorbance was read at 562 nm, and protein concentration calculated from the standard curve. Oxygen consumption was expressed as nMol O_2_/min/mg of cellular protein.

### Isolation of murine cardiac mitochondria

Hearts were extracted into ice‐cold CP1 buffer (KCl 100 mmol/L, MOPS 50 mmol/L, MgSO_4_ 5 mmol/L, EGTA 1 mmol/L, ATP 1 mmol/L, pH 7.4 with KOH). The blood was removed by rinsing, tissue weighed, and sliced before being homogenized in a dounce with a teflon pestle (1 mL of CP1 buffer per 100 mg tissue). After 16 strokes with the dounce, the homogenate was transferred to an Eppendorff tube and incubated with 100 *μ*L trypsin (stock concentration 5 mg/mL) per mL of CP1 buffer for 10 min on ice. Homogenates were subsequently transferred to the dounce and combined with equal volumes of CP2 (CP1 with 2 mg/mL fatty acid free BSA) and homogenization completed by several further dounce strokes. Samples were spun at 1000 *g* for 10 min at 4°C, the supernatant transferred to fresh Eppendorffs and respun at 5200 *g* for 10 min. The pellets were collected and resuspended in CP2 buffer before being respun at 5200 *g* for 10 min. Pellets were finally resuspended in 200 *μ*L KME buffer (CP1 buffer minus the ATP) and protein concentration quantified by Bradford assay.

### Measurement of mitochondrial respiration

Mitochondrial respiration was assessed by measuring oxygen consumption in an Oxytherm (Hansatech, UK) with a magnetic stirrer at 25°C. After quantification of mitochondrial protein concentration, all mitochondrial samples were normalized to a standard protein concentration of 5 *μ*g/*μ*L. Seventy *μ*L of mitochondria (equivalent to 350 *μ*L of mitochondrial protein) were added to 230 *μ*L of Miro 5 buffer (in mmol/L, EGTA 0.5, MgCl_2_.6H_2_0 3, K‐lactobionate 60, Taruine 20, KH_2_PO_4_ 10, HEPES 20, Sucrose 110, fatty acid free BSA 1 g/L, pH 7.0 with KOH) with 10 mmol/L glutamate, 10 mmol/L malate, 10 mmol/L Na pyruvate to stimulate Complex I respiration (State 2 respiration). State 3 respiration was stimulated by the addition of 0.5 mmol/L ADP, leak respiration through the addition of 0.25 mmol/L oligomycin, and maximal uncoupled respiration with sequential additions of 2 μmol/L FCCP. Finally, 5 μmol/L antimycin A was added to inhibit all respiration and assess nonmitochondrial loss of O_2_ from the chamber. Oxygen consumption was expressed as nMol O_2_/min/mg of mitochondrial protein.

### Statistical analysis

Results were analyzed using an unpaired *t*‐test or an one‐way analysis of variance followed by a Tukey's posttest where appropriate. Statistical significance was achieved when *P* < 0.05.

## Results

### Effects of blebbistatin and BDM supplementation on cardiomyocyte viability

**Figure 1:**
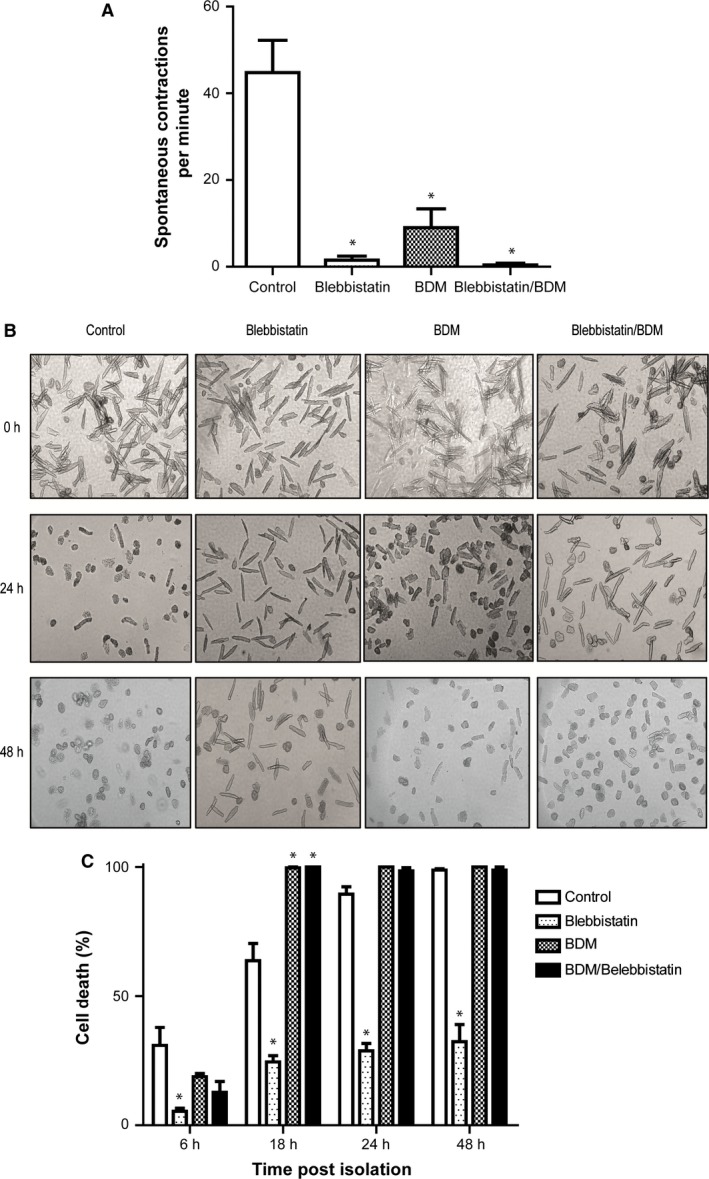
Viability of isolated mouse ventricular myocytes in M199 culture media unsupplemented or supplemented BDM with blebbistatin or BDM and blebbistatin. (A) The frequency of spontaneous contractions measured per minute immediately after plating is significantly reduced by culture with blebbistatin, BDM, or blebbistatin and BDM in combination. *versus control *P* < 0.05. (B) The time course of cell viability of isolated ventricular mouse myocytes in culture at 2, 24, and 48 h postplating (representative images). Supplementation with blebbistatin alone maintains cell viability to a greater extent that culture with BDM, blebbistatin and BDM in combination, or without any supplementation at both 24 and 48 h. (C) Viability of myocytes assessed by PI staining at 6, 18, 24, and 48 h after plating. Supplementation with blebbistatin keeps a greater proportion of isolated mouse myocytes alive throughout the 48 h post isolation. *versus control *P* < 0.05.

As expected blebbistatin and/or BDM supplementation significantly inhibited spontaneous cardiomyocyte contraction (Fig. 1A). Control isolated adult murine ventricular cardiomyocytes cultured in the absence of blebbistatin or BDM underwent hypercontracture and cell death within 24 h after plating (Fig. 1B and C). (Spontaneous contractions were counted as cells which returned immediately to their original length, while under hypercontracture, myocytes failed to return to their original morphology). However, those cells which were supplemented with blebbistatin had better cell viability at all time points up to and including 48 h following plating (blebbistatin – 32 ± 6.6% cell death vs. Control 98 ± 0.4% cell death after 48 h). Although culturing with BDM alone significantly reduced spontaneous contractions, cell viability was not significantly improved after 6 h when compared to control (BDM – 18.8 ± 1.2% cell death vs. Control 30.9 ± 6.9% cell death), but by 18 h it increased cell death when compared to control (BDM – 99.7 ± 0.2% cell death vs. Control 63.7 ± 6.6% cell death) (Fig. 1B and C), suggesting a detrimental effect with BDM on cell viability. Culturing with both BDM and blebbistatin blunted the beneficial effect on cellular viability observed with blebbistatin alone at 6 h and subsequent time points (Fig. 1B and C).

### BDM inhibits cellular respiration

**Figure 2:**
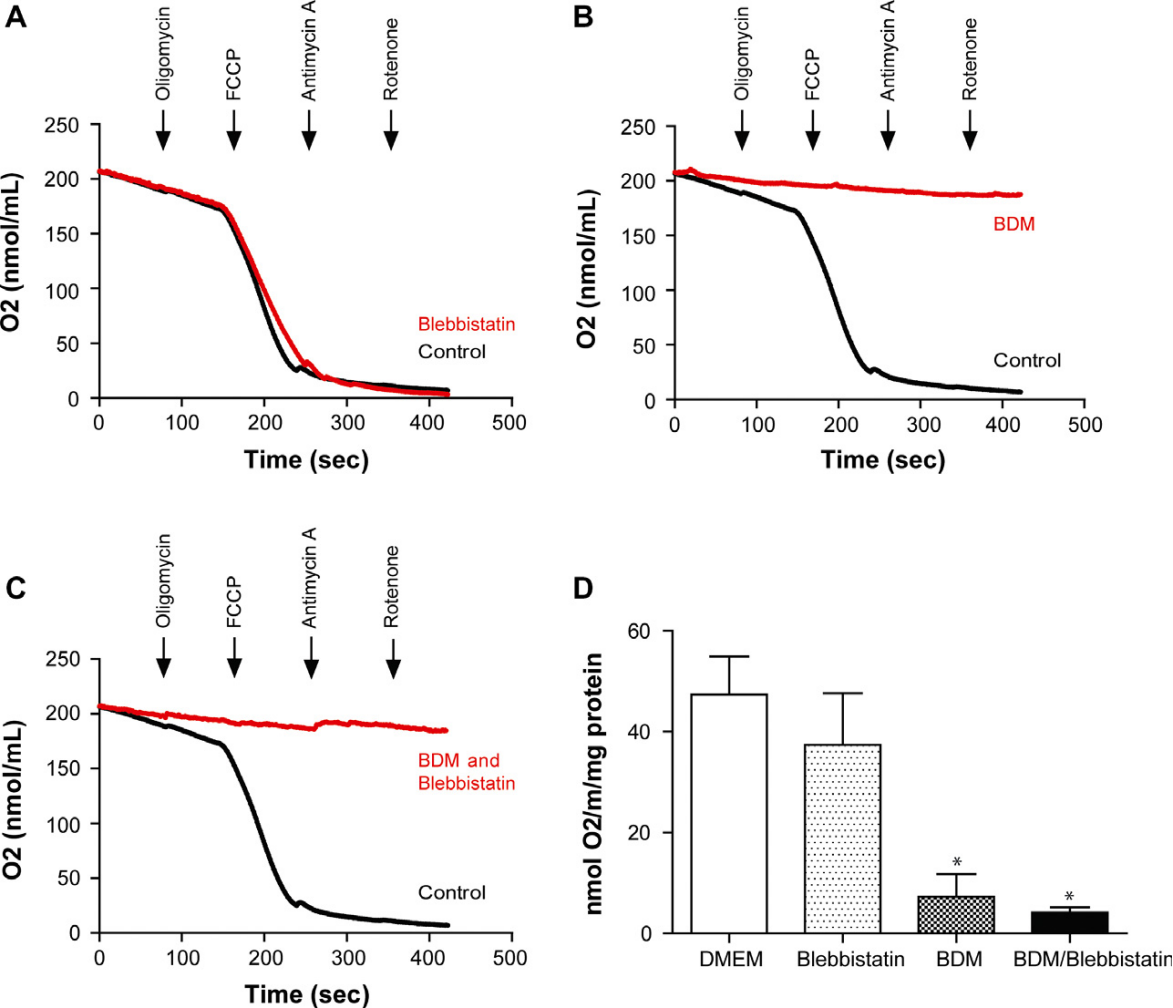
Whole cell respiration is significantly impaired in isolated adult murine ventricular cardiomyocytes when supplemented with BDM. Myocytes were either untreated or treated with blebbistatin or BDM or blebbistatin and BDM in combination prior to respiration measurements in the Oxytherm Chamber. The addition of oligomycin was used to assess leak respiration, FCCP to assess maximal respiration, and antimycin A/rotenone used to inhibit all mitochondrial oxygen consumption. Representative trace demonstrating that oxygen consumption was (A) unaffected in cells cultured with supplementary blebbistatin, (B) significantly impaired in cells cultured with supplementary BDM, and (C) significantly impaired in cells cultured with supplementary BDM and blebbistatin in combination. (D) Maximal oxygen consumption stimulated by FCCP was significantly decreased in cells cultured with supplementary BDM alone or with blebbistatin and BDM in combination, when compared to cells which were supplemented with blebbistatin alone or control. Values were calculated by dividing the maximal rate of FCCP‐stimulated oxygen consumption by the amount of myocytes protein contained in the chamber. *versus control *P* < 0.05.

In order to investigate the potential mechanisms underlying the detrimental effects of BDM supplementation on cell viability, we investigated the effect of BDM and/or blebbistatin on cellular respiration (Fig. 2A–D). Following a period of stabilization, the addition of oligomycin, to inhibit ATP synthase (for the assessment of mitochondrial respiration leak) had no effect on mitochondrial respiration rate indicating that either the oligomycin was ineffective and unable to enter the mitochondria to inhibit the ATP synthase, or that the initial respiration measured was indeed independent of the ATP synthase, and thus due to electron leak through the mitochondrial respiratory complexes. It has been suggested that the inability of oligomycin to reduce mitochondrial oxygen consumption is due to the quiescence of the cells. When the cells are not contracting, the energy demands low enough to be met by glycolysis (which is independent of the mitochondria), and thus oligomycin inhibits an ATP synthase which is already inactive (Readnower et al. 2012).

To investigate the mitochondrial respiratory reserve of isolated cells, the H^+^ ionophore, FCCP, was added sequentially through repeated drug additions, until maximal respiration was stimulated. In both control and blebbistatin ‐treated cells, FCCP increased oxygen consumption to a similar extent, indicating that the mitochondria were able to respire and meet the increased demand for electron transfer. However, in cells treated with BDM alone, or BDM and blebbistatin in combination, FCCP had no effect, indicating that mitochondrial respiration was inhibited by BDM.

Antimycin A (an inhibitor of Complex III) inhibited mitochondrial respiration in both the control and blebbistatin ‐treated samples, indicating that electrons were able to flow through the electron transport chain (ETC.) in these samples. In the samples treated with BDM alone, and BDM and blebbistatin in combination, antimycin A had little effect, indicating that mitochondrial respiration was already inhibited at some point along the ETC. by BDM.

### BDM directly inhibits mitochondrial respiration

To investigate whether the inhibitory effect of BDM supplementation on cellular respiration was a direct effect on mitochondrial respiratory function, oxygen consumption was measured in isolated cardiac mitochondria in the presence of blebbistatin and/or BDM.

**Figure 3:**
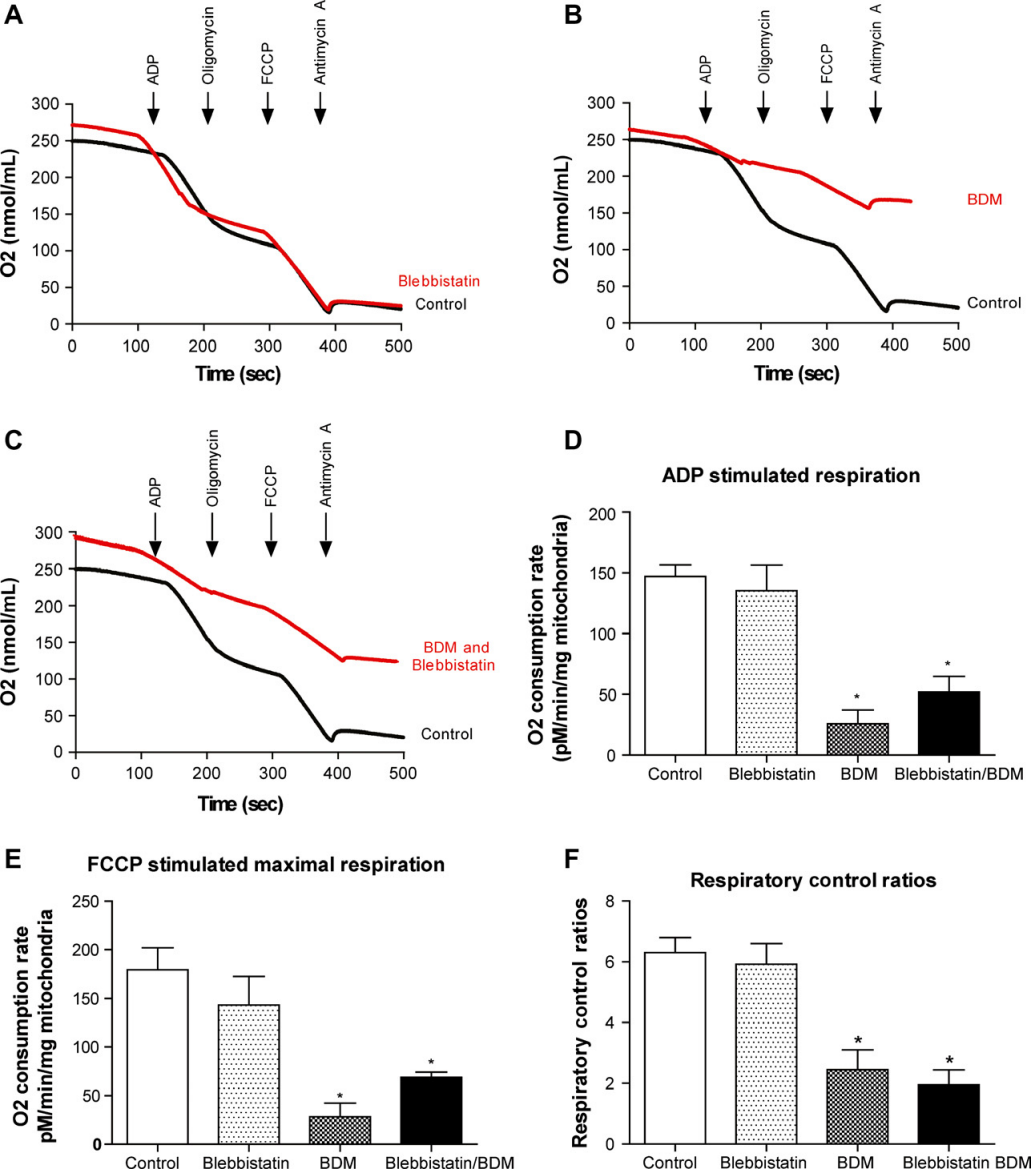
Mitochondrial respiration is significantly impaired by supplementing with BDM. Respiration was measured in isolated cardiac mitochondria either untreated or treated with blebbistatin or BDM or blebbistatin and BDM in combination. Coupled respiration was assessed through the addition of ADP and leak respiration through the addition of oligomycin. Maximal respiration was measured through the addition of FCCP before mitochondrial respiration was inhibited through the addition of antimycin A. Representative trace demonstrating that mitochondrial respiration is (A) unaffected by treatment with blebbistatin, (B) significantly impaired by the addition of BDM, and (C) significantly impaired by treatment with both blebbistatin and BDM. (D) ADP‐stimulated (coupled) respiration is significantly inhibited when mitochondria are incubated with BDM alone or BDM and blebbistatin in combination (*P > 0.05 vs. control, one‐way ANOVA with Tukey's posttest). Treatment with blebbistatin alone had no effect on ADP‐stimulated (coupled) respiration. (E) Treated with either BDM or blebbistatin and BDM in combination, significantly reduced the maximal oxygen consumption stimulated by FCCP, compared to control mitochondria (*P > 0.05 vs. control, one‐way ANOVA with Tukey's posttest). Treatment with blebbistatin alone did not alter mitochondrial maximal respiratory capacity, being similar to control. (F) Respiration control ratios (ADP‐independent respiration/ADP‐dependent respiration) were significantly lower in mitochondria treated with BDM alone or both BDM and blebbistatin in combination (*P < 0.05 vs. control, one‐way ANOVA with Tukey's posttest). Treatment with blebbistatin alone did not alter the respiration control ratios, being similar to control.

The addition of blebbistatin had no effect on mitochondrial respiration when compared to control (Fig. 3). Under these conditions, mitochondrial respiration was stimulated by both coupled (ADP) and uncoupled (FCCP) agonists, and to the same degree. However, mitochondria treated with BDM alone or BDM and blebbistatin in combination were unable to increase their respiratory rate in response to either ADP or FCCP, indicating that respiration was again inhibited to a basal leak level. The inhibition of mitochondrial respiration by BDM was especially striking when comparing the respiratory control ratios (RCR, initial Complex I respiration/ADP‐stimulated respiration). RCR values for healthy mitochondria are expected to be between 6 and 8 (Silva and Oliveira 2012), which are observed for both the control (6.3 ± 0.4) and blebbistatin ‐treated (5.9 ± 0.6) mitochondria. However, the mitochondria treated with BDM alone or BDM and blebbistatin in combination only achieved RCRs of 2.4 (±0.4) and 1.9 (±0.6), indicating highly uncoupled and dysfunctional mitochondria. A similar trend was observed when maximal respiration was stimulated with the addition of FCCP. FCCP stimulated a large increase, and maximal respiration in both the control and blebbistatin ‐treated mitochondria. In mitochondria treated with BDM alone or BDM and blebbistatin in combination, FCCP‐stimulated mitochondrial respiration was significantly lower, indicating a direct inhibitory effect of BDM on the mitochondria.

## Discussion

The main findings of this study are as follows: (1) supplementation with blebbistatin prolonged the viability of isolated adult murine ventricular cardiomyocytes without affecting mitochondrial respiration; (2) supplementation with BDM was detrimental to both cellular viability and mitochondrial respiration.

In the results presented here, we show that culturing cells with blebbistatin significantly improves cell viability in terms of both number and duration. This is important since a range of experiments are conducted over long time periods (such as viral transfections), and require a large number of healthy cells (western blots etc.). Similar results were reported by Kabeva et al., who also reported that blebbistatin, as the more specific myosin II ATPase inhibitor, is superior to BDM as a culture supplement in terms of both cell viability, and transfection efficiency (Kabaeva et al. 2008).

BDM has been reported to have several off‐target effects, including altered L‐type Ca^2+^ channel activity, SR Ca^2+^ load (Gwathmey et al. 1991) and action potential characteristics and conduction velocity (Kettlewell et al. 2004; Lou et al. 2012). In the current study, we report that BDM also acts as a potent inhibitor of mitochondrial respiration. This, in part, may explain why cell viability is reduced when supplemented with BDM. While the exact mechanism of the inhibitory effect is beyond the scope of this study, our data have demonstrated that this effect is a direct effect on mitochondrial respiration, possibly at the level of the electron transport chain. Our observation made in mouse cardiac mitochondria and cardiac myocytes is in agreement with other studies performed on rat and guinea pig hearts, which also report that BDM inhibits mitochondrial respiration at Complex I (Hebisch et al. 1993; Scaduto and Grotyohann 2000). This is an important consideration since cardioprotective drug targets may reside in the mitochondria (Walters et al. 2012; Hernandez‐Resendiz et al. 2013). If the basic physiological function of the mitochondria is inhibited, the effect of the drug and pathways will be altered, leading to conflicting and misleading results. While we report a detrimental effect upon survival, others report that BDM is able to protect the heart against cardiac ischemia–reperfusion (Garcia‐Dorado et al. 1992). However, a common mechanism (metabolic inhibition) can explain these apparent contradictory outcomes. Recent studies suggest that mitochondrial metabolism in the absence of oxygen leads to an accumulation of succinate, and upon reperfusion, this generates reactive oxygen species, which are the main source of damage to the heart (Chouchani et al. 2014). An earlier study from the same group (Chouchani et al. 2013) shows that reversible inhibition of Complex I through the addition of MitoSNO upon reperfusion is protective. By inhibiting Complex I, the pathway through which electrons from succinate flow in reverse (reverse electron transfer) to Complex I to generate ROS, is inhibited, protecting the heart. Thus, the inhibition of mitochondrial metabolism (through Complex I inhibition; Scaduto and Grotyohann 2000) with BDM during ischemia, and into reperfusion, may work through a similar mechanism. With BDM inhibiting mitochondrial respiration, the burst of reactive oxygen species upon reperfusion will be dampened, protecting the heart against I‐R damage. Importantly, we advocate that culture of mouse cardiac myocytes with BDM is detrimental.

Notably, we have found that blebbistatin increased cell viability without having any effect on mitochondrial respiration. Blebbistatin has also been reported to not affect many other important cardiac characteristics, such as the cardiac action potential (Fedorov et al. 2007; Lou et al. 2012), L‐type Ca^2+^ channel activity (Dou et al. 2007), or Ca^2+^ transient propagation (Farman et al. 2008). It is worth noting that an interaction between blebbistatin and Fluo‐4 has been reported, which prohibits the use of this Ca^2+^ indicator (Farman et al. 2008).

In summary, when culturing isolated adult murine ventricular cardiomyocytes supplementation with blebbistatin is more effective at prolonging cell viability than BDM. Furthermore, we show for the first time that BDM inhibits mitochondrial respiration and adversely affects cell viability and should not be used when culturing murine cardiomyocytes.

## Conflict of Interest

None declared.
